# Multiview deep learning networks based on automated breast volume scanner images for identifying breast cancer in BI-RADS 4

**DOI:** 10.3389/fonc.2024.1399296

**Published:** 2024-09-06

**Authors:** Yini Li, Cao Li, Tao Yang, Lingzhi Chen, Mingquan Huang, Lu Yang, Shuxian Zhou, Huaqing Liu, Jizhu Xia, Shijie Wang

**Affiliations:** ^1^ Department of Ultrasound, The Affiliated Hospital of Southwest Medical University, Sichuan, China; ^2^ Department of Radiology, The Affiliated Hospital of Southwest Medical University, Sichuan, China; ^3^ Department of Breast Surgery, The Affiliated Hospital of Southwest Medical University, Sichuan, China; ^4^ Artificial Intelligence Innovation Center, Research Institute of Tsinghua, Guangdong, China

**Keywords:** BI-RADS 4, deep learning, breast cancer, automated breast ultrasound, segmentation

## Abstract

**Objectives:**

To develop and validate a deep learning (DL) based automatic segmentation and classification system to classify benign and malignant BI-RADS 4 lesions imaged with ABVS.

**Methods:**

From May to December 2020, patients with BI-RADS 4 lesions from Centre 1 and Centre 2 were retrospectively enrolled and divided into a training set (Centre 1) and an independent test set (Centre 2). All included patients underwent an ABVS examination within one week before the biopsy. A two-stage DL framework consisting of an automatic segmentation module and an automatic classification module was developed. The preprocessed ABVS images were input into the segmentation module for BI-RADS 4 lesion segmentation. The classification model was constructed to extract features and output the probability of malignancy. The diagnostic performances among different ABVS views (axial, sagittal, coronal, and multi-view) and DL architectures (Inception-v3, ResNet 50, and MobileNet) were compared.

**Results:**

A total of 251 BI-RADS 4 lesions from 216 patients were included (178 in the training set and 73 in the independent test set). The average Dice coefficient, precision, and recall of the segmentation module in the test set were 0.817 ± 0.142, 0.903 ± 0.183, and 0.886 ± 0.187, respectively. The DL model based on multiview ABVS images and Inception-v3 achieved the best performance, with an AUC, sensitivity, specificity, PPV, and NPV of 0.949 (95% CI: 0.945-0.953), 82.14%, 95.56%, 92.00%, and 89.58%, respectively, in the test set.

**Conclusions:**

The developed multiview DL model enables automatic segmentation and classification of BI-RADS 4 lesions in ABVS images.

## 1 Introduction

Breast cancer has become the most prevalent cancer worldwide, with 2.3 million new cases resulting in 665,684 deaths in 2022 (1). Accurate identification and timely treatment are effective measures to reduce its mortality. Breast Imaging Reporting and Data System (BI-RADS) category 4 lesions (2) are suspected to be malignant lesions (2%~95% likelihood) and are recommended for biopsies, which results in more than 67.0% of benign lesions receiving biopsies (3–6). This may lead to unnecessary anxiety and invasive examination-related complications, such as pain, infection, and needle track seeding, in patients as well as increase the burden to the healthcare system (7). Therefore, a noninvasive method for identifying malignant BI-RADS 4 lesions and reducing unnecessary biopsies is an urgent issue in current precision medicine.

Ultrasound (US) is not inferior to mammography for screening for breast cancer and has a sensitivity of up to 90% in dense breasts with safety and low cost (8, 9). The automated breast volume scanner (ABVS) is a novel breast ultrasound imaging technique that overcomes many of the limitations of traditional US, provides a three-dimensional (3D) representation of breast tissue, and allows image reformatting in three planes (axial, sagittal and coronal) (10). The ABVS’ unique coronal images provide an intuitive view of the lesions and their relationships with neighboring catheters and surrounding tissues. The retraction phenomenon, characterized by a perinodal stripe of hypoechoic and hyperechoic radial extension, is a unique sign on the coronal plane for malignant breast tumors with a high specificity (91.1%~100%) (11, 12). However, the large amount of ABVS image data is a significant challenge for radiologists.

Deep learning (DL) is a subfield of artificial intelligence (AI), and its emergence has increased interest in automated detection and diagnostic tools in medicine (13). DL has achieved state-of-the-art performance in feature recognition and classification in several modalities, including magnetic resonance imaging (MRI), computed tomography (CT), X-ray and US (14–17). Recent studies have shown that DL methods using ABVS images also have enormous potential in breast cancer (18–20). Wang et al. (18) proposed a DL method that adopted a modified Inception-v3 architecture to extract effective features from ABVS images to distinguish between benign and malignant breast lesions with an area under the curve (AUC), sensitivity, and specificity of 0.945, 0.886, and 0.876, respectively. However, most of these studies were designed as proof-of-concept or technical feasibility studies without a thorough external validation of real-world clinical performance (19, 20). To our knowledge, no studies have investigated the use of DL methods based on ABVS images to distinguish between benign and malignant BI-RADS 4 lesions.

Therefore, we attempted to develop a DL model based on ABVS images with automatic segmentation and classification capabilities, and to explore its performance in identifying benign and malignant BI-RADS 4 lesions and in reducing unnecessary biopsies. In addition, since ABVS images can be visualized in axial, sagittal and coronal views, we further compared the DL models based on the use of single or multiple views.

## 2 Materials and methods

This retrospective study was approved by the Institutional Review Board of the Affiliated Hospital of Southwest Medical University (KY2020163) and was conducted following the Declaration of Helsinki guidelines. All participating subjects were informed and voluntarily signed informed consent forms.

### 2.1 Patients and data collection

From 1 May to 31 December, 2020, consecutive patients with BI-RADS 4 lesions on US who were scheduled for biopsies at the Affiliated Hospital of Southwest Medical University (Centre 1, the training set) and Guangdong Provincial Hospital of Traditional Chinese Medicine (Centre 2, the independent test set) were invited to participate in this study. Further selection was performed according to the following inclusion and exclusion criteria.

The inclusion criteria were as follows: (1) age≥18 years; (2) BI-RADS 4 lesions identified following the 2013 edition of the BI-RADS guidelines (21) by two senior radiologists (>10 years of breast US experience) at both centers; and (3) completion of the ABVS examination within one week before biopsy. The exclusion criteria were as follows: (1) patients who were breastfeeding or had mastitis or breaks in the affected breast; (2) patients who had undergone previous invasive procedures for the lesion; (3) patients with poor-quality images; and (4) patients who lacked definitive pathologic findings. Patients with more than one BI-RADS 4 lesion were included separately.

Clinical data included age, menopausal status, history of oral contraceptive use and smoking history, alcohol consumption level, and family history of breast or ovarian cancer. The patient’s breast density was classified as type A-D according to the mammographic BI-RADS guidelines. The characteristics of the lesions, including the lesion size, location (left or right), shape (regular or irregular), orientation (parallel or nonparallel), posterior echogenicity (enhancement, shadowing, mixed pattern, or absence of posterior echogenicity), internal echogenicity (hypoechoic, hyperechoic, or mixed echogenicity), and calcification (present or absent), were recorded.

### 2.2 ABVS examinations

**Figure 1:**
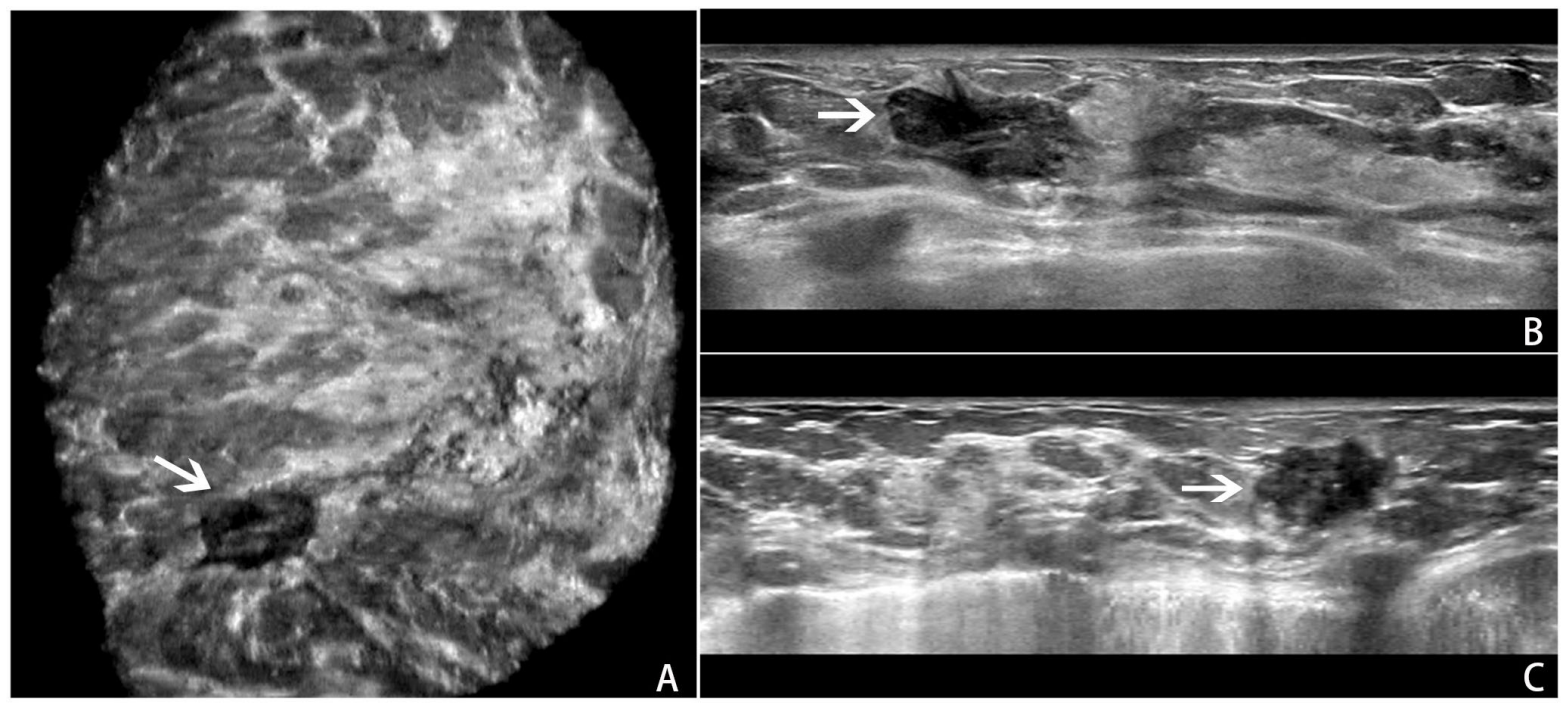
An example of a BI-RADS 4 lesion on ABVS images.ABVS images of the largest sections of a lesion in the coronal **(A)**, axial **(B)**, and sagittal **(C)** planes.

All ABVS examinations were performed by the Acuson S2000 ABVS (Siemens, Germany) ultrasound systems with the 14L5BV probe (5–14 MHz) by two technicians (with 6 months of ABVS training experience). For more details on the ABVS examination, see Kim et al (22). After the examination, axial ABVS images were sent to a dedicated workstation, and the sagittal and coronal images were reconstructed automatically. Finally, the axial, sagittal, and coronal ABVS images showing the largest lesions were selected for further segmentation and classification. An example is shown in **Figure 1**.

Within one week after the ABVS examination, a US-guided core-needle biopsy was performed by experienced US doctors. In accordance with the standard biopsy procedure, four to eight samples per lesion were acquired via an automatic biopsy gun with a 14G or 16G needle. The specimens were analyzed and diagnosed by breast pathologists (>10 years of experience), according to the World Health Organization’s standards for breast tumor classification (23). For lesions with unclear diagnoses by puncture, histopathologic diagnosis after surgical removal was used as the reference standard.

### 2.3 DL Framework and models

**Figure 2:**
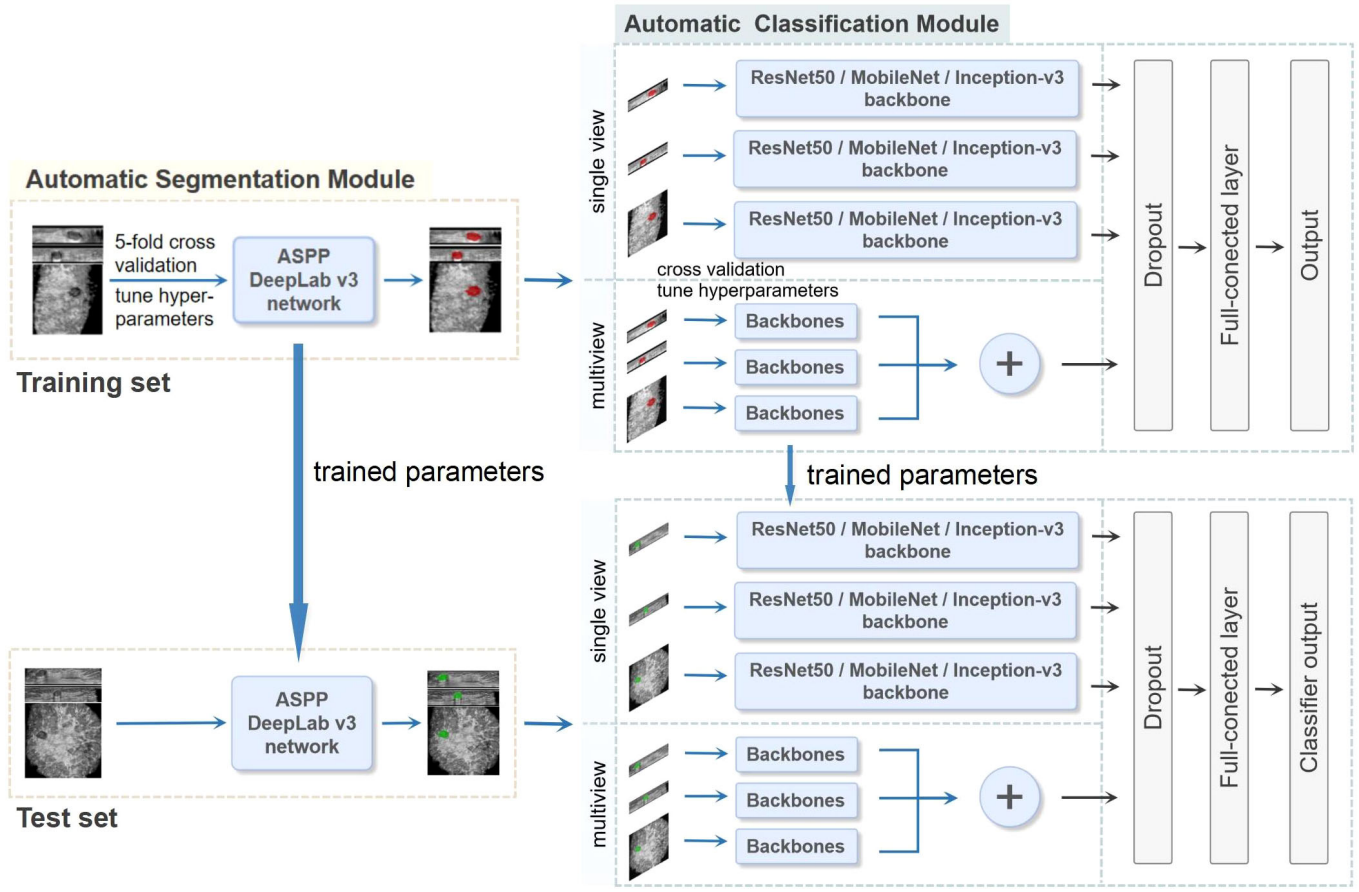
The whole pipeline of the deep learning (DL) model. The illustration shows the image input and the main processing stages for the two-stage DL framework model, which consists of an automatic segmentation module and an automatic classification module. The preprocessed ABVS images were used as input for the segmentation module for segmenting lesions. The classification module was constructed on the basis of single-view (axial, sagittal, and coronal) and multiview (combined axial, sagittal, and coronal) images, as well as different backbone networks (ResNet50, MobileNet and Inception-v3), and outputs the probability of malignancy. In the multiview model, each view of the input images corresponds to a backbone network branch, and three branches are concatenated to form the total feature vector. Five-fold cross-validation was utilized on the training set to choose the hyperparameters. The test set was used to evaluate the final performance.

Centers 1 and 2 were divided into a training set and an independent test set, respectively. We utilized five-fold cross-validation on the training set to optimize the parameters of the models and guide the choice of hyperparameters. The test set was used to evaluate the final model performance independently. A two-stage DL framework consisting of an automatic segmentation module and an automatic classification module was developed. First, the preprocessed ABVS images were input into the automatic segmentation module for BI-RADS 4 lesion segmentation. Patches were created as the input to the classifier. The classification model was subsequently constructed via convolutional neural networks (CNNs) to automatically extract the features of the lesions and output the probability of malignancy. The overall process is described in detail below and the whole pipeline of the DL model is shown in **Figure 2**. Finally, we visualized and analyzed the prediction results of the DL model.

#### 2.3.1 Image preprocessing and automatic segmentation module

Histogram equalization and median filtering were used to remove noise and enhance the images. The black boxes in the ABVS images were cropped using the Sobel operator (24). Online data augmentation was performed for the ABVS images in the training set during the training period. The augmented image pixels were normalized and input into the ImageNet dataset for pretraining.

The DeepLab-V3 algorithm introduced by Google was used to build the automatic image segmentation module. DeepLab-V3 uses the atrous spatial pyramid pooling (ASPP) structure to expand the receptive field, mining context information, and the improved Xception module to reduce the number of parameters and achieve the best effect of the current segmentation network.

#### 2.3.2 Automatic classification module

**Figure 3:**
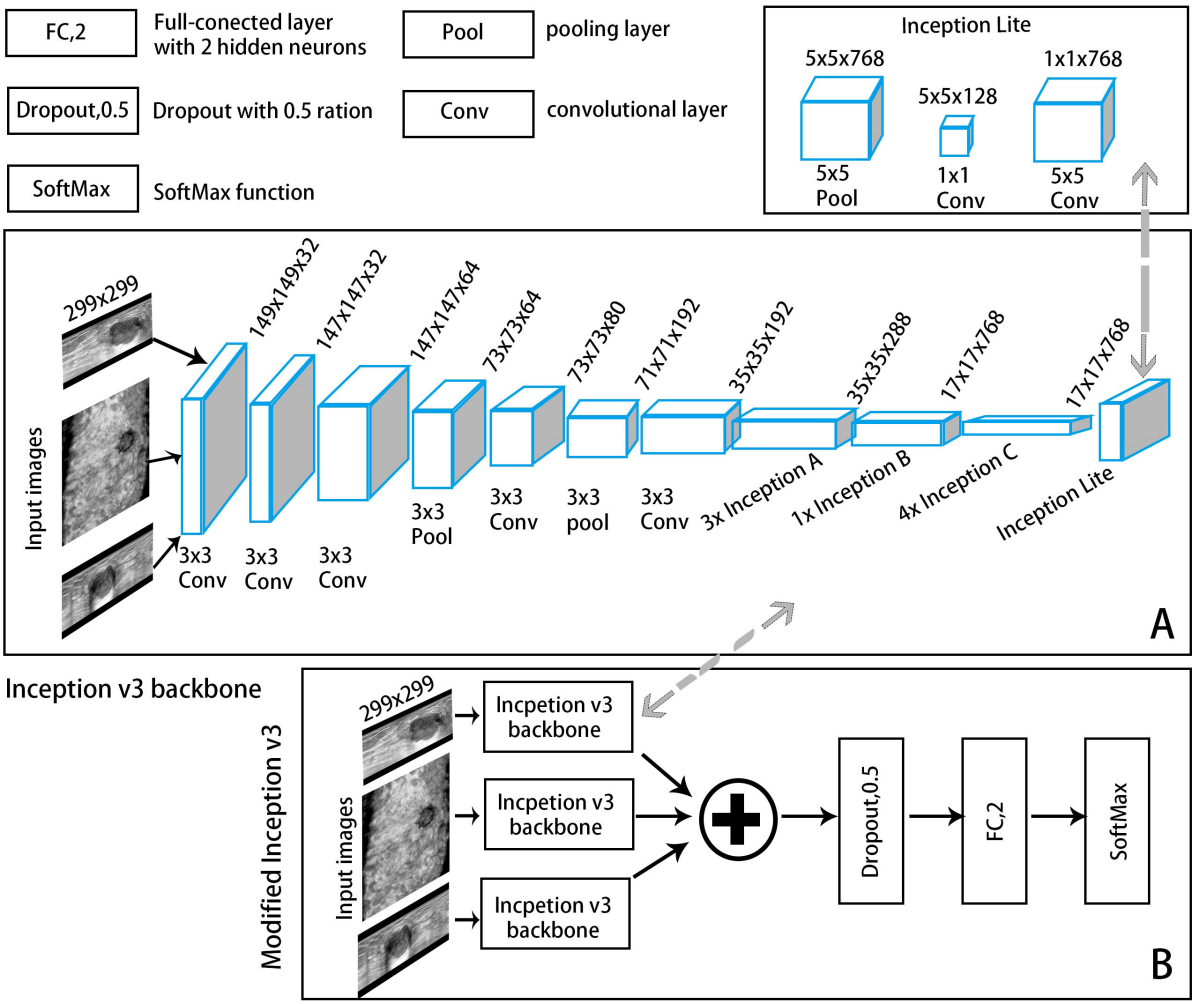
A convolutional neural network (CNN) framework example of an automatic classification model with Inception-v3 as the backbone network. **(A)** The backbone network: The input ABVS image in three views (axial, sagittal, and coronal) passed six convolutional layers and one average pooling layer, followed by three Inception A modules, one Inception B module, four Inception C modules and one Inception Lite module as defined in this study. The Inception Lite module consists of an average pooling layer in tandem with two convolutional modules of different kernel sizes. **(B)** The modified version of Inception-v3.

The segmented images of the lesion and its surrounding area were as patches to input to the classification module to extract features and automatically output the probability of malignancy. For the reasons that manually labelling masks has a certain degree of subjectivity; the segmentation results of the segmentation model also have certain biases; and the differences between the lesion area and nearby normal tissues may help AI classify more accurately. To construct the optimal DL model, we explored the performances of CNN models based on single-view (axial, sagittal, and coronal) and multiview (combined axial, sagittal, and coronal) images, as well as different backbone networks (ResNet50, MobileNet, and Inception-v3) in differentiating benign and malignant BI-RADS 4 lesions. Transfer learning was applied to ensure a strong feature extraction capability. Because of the limited number of samples, pretrained knowledge was effectively applied to a specific task from a mega database such as ImageNet, and the model was then retrained using a small amount of data, which could achieve satisfactory results (25). Each model was fine-tuned on the dataset of ABVS images to reduce overfitting. The convolutional structure was used as the backbone network, consisting of multiple convolutional layers, average pooling layers, and convolutional modules in series for feature extraction. In the multiview models, each view of the input images corresponds to a backbone network branch, and three branches are concatenated to form the total feature vector. A CNN framework example of Inception-v3 is shown in **Figure 3**. A dropout layer (with deactivation rate of 0.5) was added behind the vector to mitigate overfitting. Finally, the fully connected layer was normalized to output the probability of malignancy of BI-RADS 4 lesions (a cut-off value of 50%).

The image preprocessing methods and DL algorithms with the parameters and software settings are detailed in **Supplementary File 1**.

#### 2.3.3 Testing and visualization of the DL model

The fine-tuned parameters were used in the segmentation and classification models of the independent test set to evaluate the effectiveness and final performance of these models. The results were analyzed and assessed by the area under the receiver operating characteristic (ROC) curve. The sensitivity, specificity, positive predictive value (PPV), and negative predictive value (NPV) were calculated at the maximum Youden index. The performance of the automatic segmentation network was evaluated via the Dice coefficient (DC). Additionally, we set a decision point in the ROC curve based on the final model where sensitivity is 100% to evaluate the value in reducing the unnecessary biopsies and this would allow no lesions to be missed.

Gradient-weighted class activation mapping (Grad-CAM) was used on the final convolutional layer of the classification model to visualize the extent of each region on the ABVS image that contributed to identifying malignant BI-RADS 4 lesions. The critical areas predicted by the model are highlighted.

### 2.4 Statistical analysis

IBM SPSS Statistics (version 26.0, IBM Corp., USA) and Python software (version 3.6.8, https://www.python.org/) were used for the statistical analysis. SPSS software was used to analyze the differences between the training and test sets and between benign and malignant lesions. Continuous variables (age and tumor size) were compared via t-tests. Categorical variables (breast density, BI-RADS 4 subclasses, and family history of breast cancer) were compared via the chi-square test.

The DC, recall, and precision were introduced to evaluate the automatic segmentation performance objectively. ROC curves were constructed to assess the classification performance and to calculate the sensitivity, specificity, PPV, NPV, and AUC. The AUCs were compared via the DeLong test. All the statistical calculations were performed with 95% confidence intervals (95% CIs). All tests were two-sided, and *P*<0.05 was considered statistically significant.

## 3 Results

### 3.1 Patient characteristics

**Figure 4:**
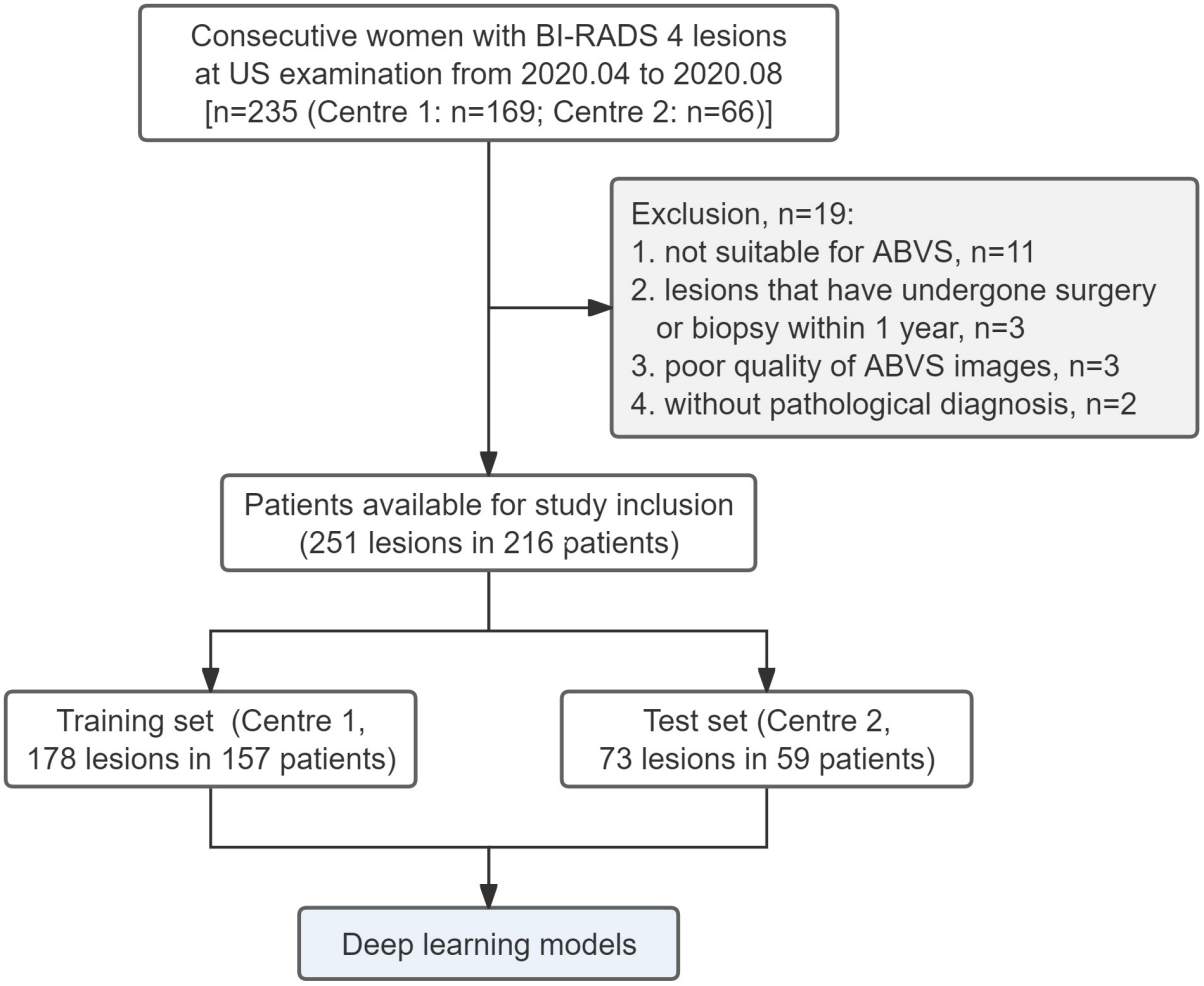
Flow chart of the study.

A total of 251 BI-RADS 4 lesions in 216 patients from two centers were included. The flow chart is shown in **Figure 4**. Among them, 178 lesions from 157 patients (mean age 49.0 ± 11.8 years) at Centre 1 were included in the training set, and 73 lesions from 59 patients (average age 46.8 ± 10.5 years) at Centre 2 were included in the independent test set. The proportions of malignant lesions between the two sets were not significantly different (45.5% *vs*. 39.7%, *P*=0.402), and there were no significant differences in patient age, lesion size, lesion location, BI-RADS 4 subclassifications, breast density, or family history (**Table 1**).

**Table 1:** Baseline data of the benign and malignant BI-RADS 4 lesions in the training and test sets.

Characteristics	Training set (n=178)			Test set (n=45)			P *
	Malignant (n=81)	Benign(n=97)	*P*	Malignant (n=29)	Benign(n=44)	*P*	
Age (years, ±)	54.6 ± 12.5	44.1 ± 9.9	<0.001	57.5 ± 9.4	45.4 ± 11.7	<0.001	0.229
Lesion size (cm, ±)*	2.3 ± 0.9	1.6 ± 0.9	<0.001	2.5 ± 1.2	1.6 ± 0.7	0.005	0.549
BI-RADS 4 category (n, %)							
4a	11(13.3%)	73(76.0%)	<0.001	2(6.9%)	35(79.5%)	<0.001	0.593
4b	16(19.3%)	20(20.0%)		4(13.8%)	9(20.5%)		
4c	54(67.4%)	4(4.0%)		23(79.3%)	0(0.0%)		
Breast density (n, %)							
A	11(13.2%)	4(4.0%)	<0.001	4(13.8%)	0(0.0%)	0.319	0.878
B	38(45.8%)	26(27.0%)		12(41.4%)	17(38.6%)		
C	26(45.8)	42(44.0%)		9(31.0%)	20(45.5%)		
D	6(7.2%)	25(25.0%)		4(13.8%)	7(15.9%)		
Menopausal status (n, %)							
Premenopausal	33(41.0%)	73(74.0%)	<0.001	6(20.7%)	29(65.9%)	0.010	0.054
Postmenopausal	48(59.0%)	24(26.0%)		23(79.3%)	15(34.1%)		
Family history (n, %)							
Yes	8(9.6%)	8(8.0%)	0.705	6(20.7%)	13(29.5%)	0.793	0.153
No	73(90.4%)	89(92.0%)		23(79.3%)	31(70.5%)		
Location of lesion (n, %)							
Left	51(62.7%)	52(55.05)	0.208	15(51.7%)	26(59.1%)	0.302	0.992
Right	30(37.3%)	45(45.0%)		14(48.2%)	18(40.9%)		

*P values between the training set and the test set.

Lesion size was defined as the maximum diameter on ABVS images. Family history referred to breast or ovarian cancer in first-degree relatives. The differences in characteristic variables (age and lesion size) between the two cohorts were compared via two-sample t-tests, whereas chi-square tests were conducted on the other variables. P<0.05.

BI-RADS, Breast Imaging Reporting and Data System.

### 3.2 Performance of the automatic segmentation module

The Dice coefficient curves (**Supplementary File 2**) for assessing segmentation performance revealed 474 (88.7%) ABVS images with DCs greater than 0.90 in the training set and 165 (75.3%) ABVS images in the test set. Example plots are provided in **Supplementary File 3**. The automatic segmentation model has the best segmentation performance in axial views with DCs, recall rates, and precisions of 0.908 ± 0.077, 0.996 ± 0.067, and 0.974 ± 0.123 in the training set and 0.890 ± 0.152, 0.972 ± 0.167, and 0.948 ± 0.198 in the test set, respectively. Among all the views, the segmentation module displayed the worst performance in the coronal views, with DC, recall and precision values of 0.784 ± 0.120, 0.825 ± 0.188, and 0.825 ± 0.188, respectively, which is still a satisfactory result. The detailed segmentation statistics of the different ABVS views in the two sets are shown in **Table 2**.

**Table 2:** Automatic segmentation results of different ABVS views in the training and test sets.

Clusters	Dice coefficient (mean ± SD)	Recall (mean ± SD)	Precision (mean ± SD)
The training set (n=534)	0.874 **±** 0.173	0.911 ± 0.185	0.893 ± 0.195
Coronal view (n=178)	0.804 **±** 0.121	0.894 ± 0.207	0.883 ± 0.216
Sagittal view (n=178)	0.824 ± 0.154	0.926 ± 0.163	0.905 ± 0.177
Axial view (n=178)	**0.908 ± 0.077**	**0.996 ± 0.067**	**0.974 ± 0.123**
The test set (n=219)	0.817 ± 0.142	0.903 ± 0.183	0.886 ± 0.187
Coronal view (n=73)	0.784 ± 0.120	0.825 ± 0.188	0.825 ± 0.188
Sagittal view (n=73)	0.801 ± 0.119	0.883 ± 0.157	0.888 ± 0.165
Axial view (n=73)	**0.890 ± 0.152**	**0.972 ± 0.167**	**0.948 ± 0.198**

SD refers to the standard deviation, and the bolded portion is the group with the best indicator.

### 3.3 Performance of the automatic classification module

The automatic classification of BI-RADS 4 lesions was performed after automatically segmenting the lesions.

**Figure 5:**
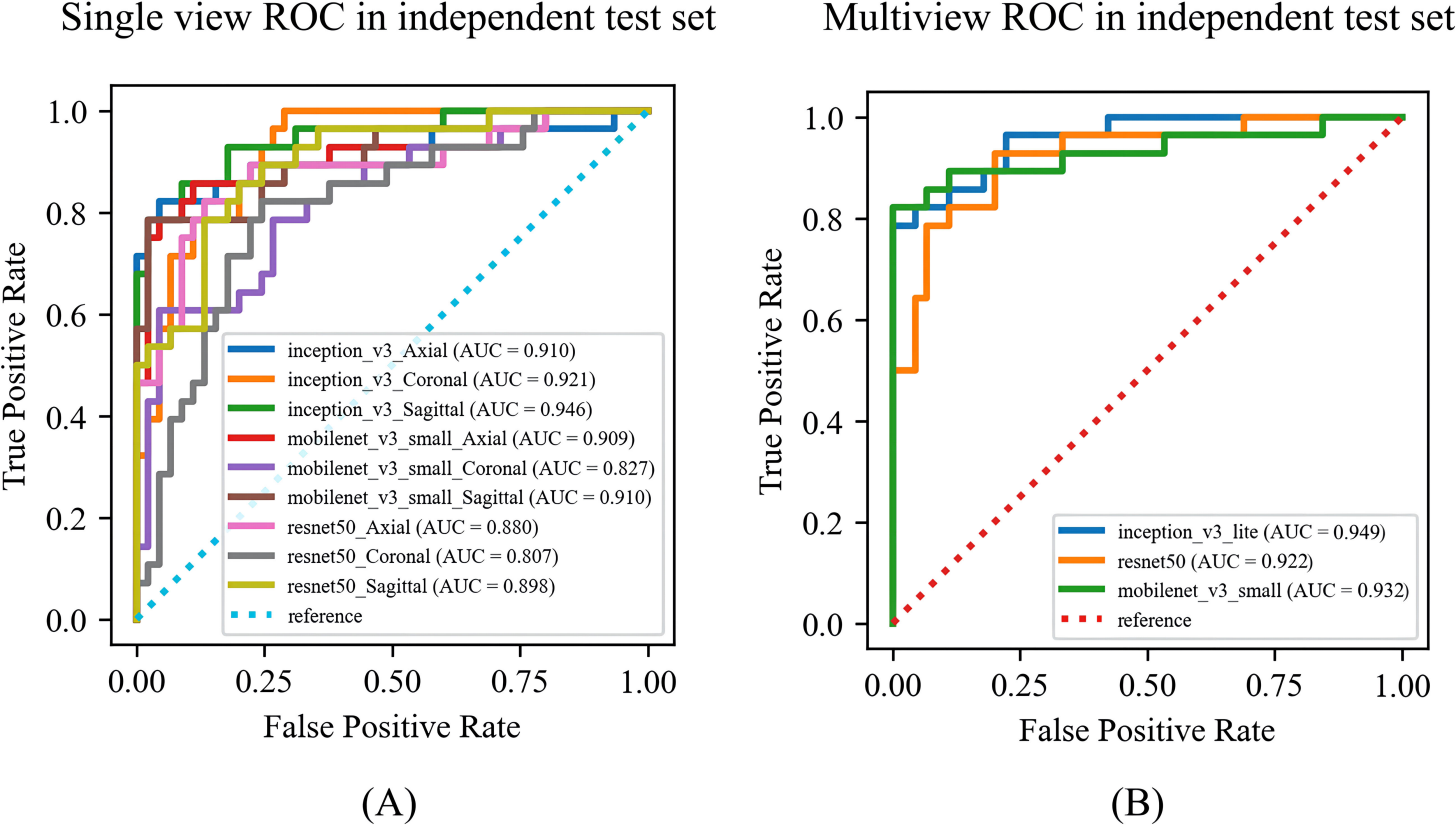
The ROC curves of the **(A)** single-view models and **(B)** multiview model based on different backbone networks (ResNet50, MobileNet, and Inception-v3) on the test set. ROC, receiver operating characteristic; AUC, area under the receiver operating characteristic curve.

Among the DL models on the various views, the multiview models had better classification performance than the single-view models in different backbone networks (ResNet50, MobileNet, and Inception-v3). Moreover, all three single-view models and the multiview model achieved the best classification performance in the Inception-v3 network on both sets. The statistics for the training set are shown in **Supplementary File 4**, and the performance results for the test set are shown in **Table 3**, **Figure 5**. Among them, the multiview model with the Inception-v3 backbone had the best performance, with an AUC, sensitivity, specificity, PPV, and NPV of 0.949 (95% CI: 0.945–0.953), 82.14%, 95.56%, 92.00%, and 89.58%, respectively. However, the coronal single-view model based on ResNet50 had the worst classification performance, with an AUC, sensitivity, specificity, PPV, and NPV of 0.807 (95% CI: 0.779–0.836), 85.71%, 57.78%, 55.81%, and 86.67%, respectively.

**Table 3:** Diagnostic performance of the single-view and multiview models based on different backbone networks (ResNet50, MobileNet, and Inception-v3) on the test set.

Backbones	View	AUC (95%CI)	Sensitivity (%)	Specificity (%)	PPV (%)	NPV (%)
ResNet50	Axial view	0.880 (0.857-0.903)	71.43	91.11	83.33	83.67
	Sagittal view	0.898 (0.880-0.915)	82.14	82.22	74.19	88.10
	Coronal view	0.807 (0.779-0.836)	85.71	57.78	55.81	86.67
	**Multiview** *	**0.922 (0.905-0.939)**	**82.14**	**84.44**	**76.67**	**88.37**
MobileNet	Axial view	0.909 (0.886-0.931)	78.57	93.33	88.00	87.50
	Sagittal view	0.910 (0.890-0.930)	78.57	97.78	95.65	88.00
	Coronal view	0.827 (0.802-0.854)	67.86	73.33	61.29	78.57
	**Multiview**	**0.933 (0.914-0.952)**	**82.14**	**93.33**	**88.46**	**89.36**
**Inception-v3**	Axial view	0.910 (0.888-0.933)	82.14	91.11	85.19	89.13
	Sagittal view	0.946 (0.932-0.961)	78.57	91.11	84.61	87.23
	Coronal view	0.921 (0.905-0.937)	85.71	77.78	70.59	89.74
	**Multiview**	**0.949 (0.945-0.953)**	**82.14**	**95.56**	**92.00**	**89.58**

*Multiview is the combination of axial, sagittal, and coronal planes. The bolded portion is the group with the best indicator.

AUC, area under the curve; CI, confidence interval; PPV, positive predictive value; NPV, negative predictive value.

### 3.4 Value in reducing unnecessary biopsies and visualizations

**Figure 6:**
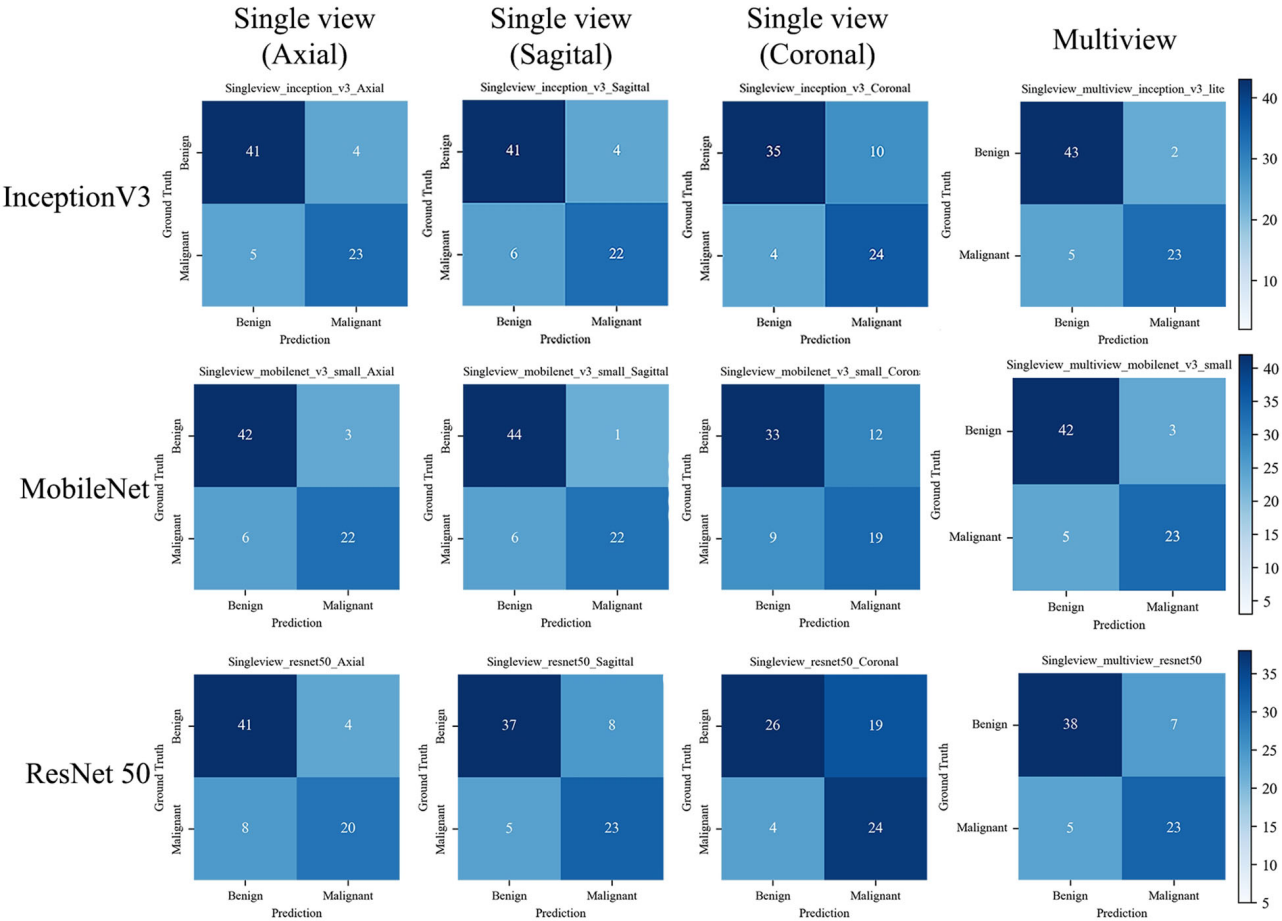
The confusion matrix of the deep learning models with the test set. The confusion matrix of the models is based on single view (axial, sagittal, and coronal) and Multiview images, as well as different backbone networks (ResNet50, MobileNet, and Inception v3) with the test set. The correct predictions are shown on the diagonal from the top left to the bottom right of each matrix.

The confusion matrix of all the DL models with the test set is shown in **Figure 6**. The Inception-v3-based multiview DL model performed the best, with a missed diagnosis rate and misdiagnosis rate of 17.85% (5/28) and 4.44% (2/45), and with the unnecessary biopsy rate reducing from 61.64% (45/73) to 8.00% (2/25) compared to the conventional US. The sagittal single-view and multiview models based on the MobileNet network achieved similar performance, with missed diagnosis rates and misdiagnosis rates of 21.43% (6/28) and 2.22% (1/45) on the sagittal view, and 17.85% (5/28) and 6.67% (3/45) on the multiview, respectively. With a decision point of 100% sensitivity in the ROC curve based on multiview Inception-v3 model, the specificity, PPV, and NPV were 58.1%, 58.9%, and 100%, respectively (The confusion matrix is shown in **Supplementary File 5**). And the unnecessary biopsy rate of it is 40.42% (19/47), which is 21.22% lower than conventional ultrasound (61.64%, 45/73) without missing any malignant lesions.

**Figure 7:**
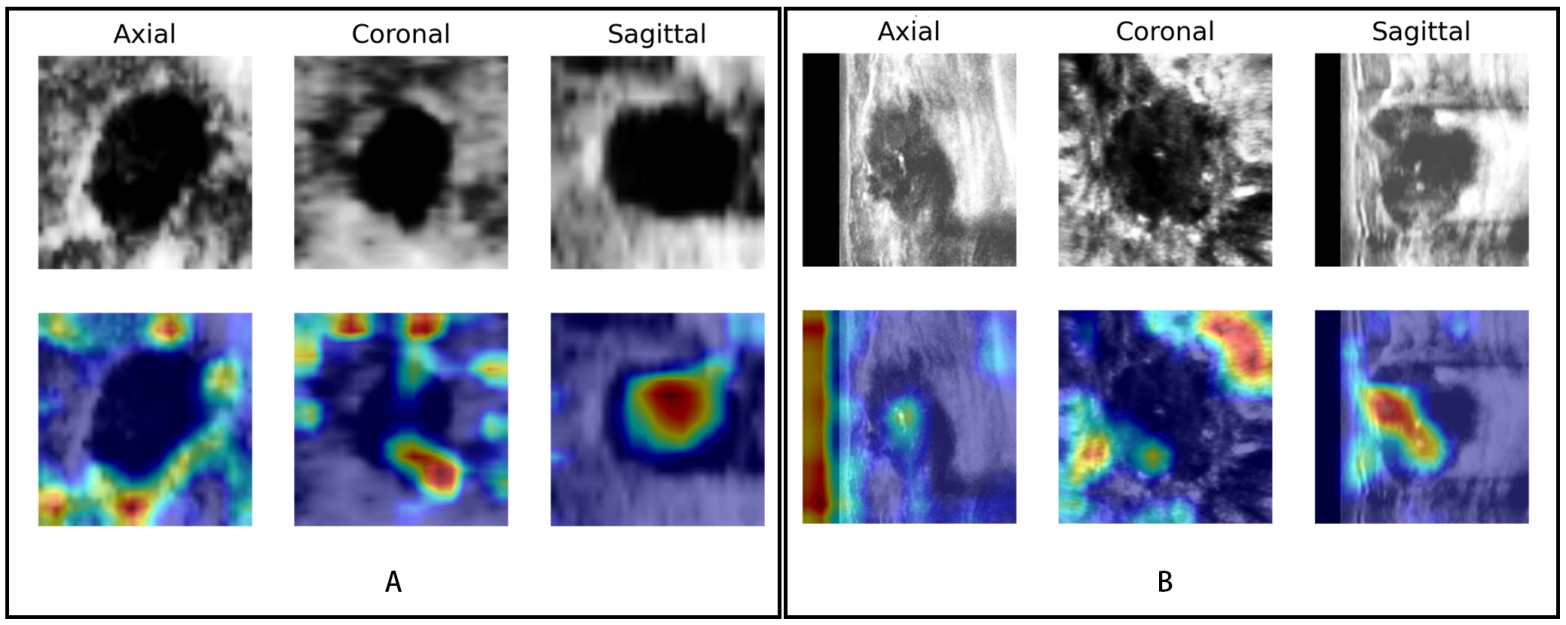
Visualization of the DL model. The upper row shows the lesions in different views (axial, coronal, and sagittal) of ABVS images; the row below shows the saliency map generated by the Grad-CAM algorithm. The red region represents a more significant weight. **(A)** A case of a benign BI-RADS 4B lesion and **(B)** a case of a malignant BI-RADS 4C lesion both showed that the DL model focused not only on the lesion itself but also on the periphery of the lesion.

The saliency map highlighted the lesion location and surrounding region, both in benign and malignant lesions (**Figure 7**). This finding indicated that the multiview DL model focused on the lesion itself and surrounding structures when categorizing BI-RADS 4 lesions.

## 4 Discussion

In this study, we developed an ABVS-based DL model with automatic segmentation and classification capabilities to explore its diagnostic performance in single-view and multiview images for identifying breast cancer in BI-RADS 4 lesions. We found that our DL model can accurately segment multiple views of ABVS images and further differentiate benign and malignant BI-RADS 4 lesions, which could reduce unnecessary invasive biopsies.

DL, a technique used in artificial intelligence, has achieved significant advances in automatic medical image analysis of breast cancer through CNNs. In addition to segmenting (26) and categorizing (27) various modalities of ultrasound images of breast cancer, DL can also predict metastasis (28) and patient prognosis (29). The BI-RADS 4 lesion is the watershed for whether to perform a biopsy, with a 5%–98% likelihood of being benign (3). Therefore, accurately differentiating the benign and malignant natures of BI-RADS 4 lesions is the key to minimizing noninvasive manipulation of breast lumps and is a pressing issue. Therefore, we used a deep learning approach to solve this problem noninvasively. To our knowledge, the development of ABVS-based DL models for the automatic segmentation and classification of BI-RADS 4 lesions, as well as the application of such an approach for reducing the possibility of biopsy, has not been reported.

This study used the DeepLab-V3 model to segment BI-RADS 4 lesions automatically, and the high DC values reflected its powerful segmentation performance. The segmentation effectiveness was the worst in the coronal plane. This may be because the artefacts caused by the nipple are extremely similar to the echogenicity of the lesion in the coronal plane, and the adipose tissue in the breast, which is morphologically similar to some breast nodules, is also in a restricted distribution in this plane. The model achieved the best segmentation performance in the axial single section, which is consistent with recent research results (30). Therefore, the DeepLab-V3-based segmentation module actually has excellent segmentation efficacy, self-learning ability, and self-adaptation for ABVS image segmentation (31, 32). Moreover, high-quality automatic segmentation lays a foundation for the subsequent standardization of feature extraction and classification accuracy (33).

ABVS can provide 3D images and reconstruct the images to axial, sagittal, and coronal views. Thus, we explored the performance of the classification module based on three single views and the combined views. Among the single-view models, the diagnostic performance on the coronal view was the lowest, whereas it was the best on the axial view, which is inconsistent with previous perceptions (34). These authors (34) suggested that the ABVS-specific coronal view maximizes the understanding of the relationship between the breast lesion and the surrounding tissues and is more conducive to identifying benign and malignant lesions. In particular, the retraction phenomenon on the coronal view has a high sensitivity (80%~89%) and specificity (96%~100%) for detecting breast cancer (11, 35). The main reasons for this contradiction may be that the classification module in this study was constructed on the basis of automatic segmentation, and the relatively poorer segmentation results on the coronal plane led to a subsequent decrease in classification performance. This finding is consistent with a recent view (36) emphasizing that accurate segmentation is a prerequisite for precise classification in DL models. This, in turn, explains the better classification performance of the axial sections. The multiview models simultaneously fused the features of the three views and demonstrated the best diagnostic performance.

Since different CNN backbone network structures may affect the classification performance of the model (37), three common backbone structures (Inception-v3, ResNet50, and MobileNet) were compared for our DL classification modelling. Inception-v3, a CNN improved by the third-generation GoogLeNet, uses multiple regular convolutional layers for feature extraction and concatenates the features as an output, which can help the model learn different-sized lesions efficiently (38). The Inception-v3-based model can handle more and richer spatial features, increase feature diversity, and reduce the number of computations, with an error rate of only 3.5% (39). It actually achieved the highest classification accuracy in the single- and multiview automatic classification models in this study, which may reduce the unnecessary biopsy rate. The optimal DL model in this study could reduce unnecessary biopsies by 53.64%, significantly outperforming previous approaches using contrast-enhanced US (40) and elastography (41), which also have more complicated procedures and rely on the experience of the examining sonographer (42). However, the DL model still had a missed diagnosis rate of 17.85%, which would delay treatment and affect the outcomes and prognoses of patients (43). Although the sagittal single-view model on the MobileNet backbone could reduce the rate of unnecessary biopsies more (57.29%), it was more likely to miss diagnosis (21.43%) than the optimal model was. Therefore, the Inception-v3-based multiview DL model was selected as the final model. On this basis, we further set a decision point with the sensitivity of 100% in the ROC curve which could reduce unnecessary biopsy rate by 21.22% without missing any lesions. However, more comprehensive research and optimization are needed before its application in the clinic. Additionally, previous studies (44, 45) have shown that both the breast lesion itself and its periphery contribute significantly to the interpretability of breast lesions, which is consistent with our Grad-CAM visualization results. To some extent, this may explain the discrimination ability of this DL model.

In conclusion, our work indicated that the ABVS-based DL model can reduce radiologists’ manual intervention through automatic segmentation and automatic classification and improve the performance of benign and malignant discrimination of BI-RADS 4 lesions. With further improvements in the model in the future, it will hopefully be promoted and applied in clinical practice, which could significantly impact the management of BI-RADS 4 lesions, reduce biopsies, and promote the development of precision medicine.

There are several limitations. First, the total number of cases of BI-RADS 4 lesions was relatively limited, which may have affected the reliability of the model. Datasets with more centers and larger samples need to be included for further validation and optimization. Second, only the largest section of each BI-RADS 4 lesion was used for analysis, which did not fully utilize the advantages of ABVS 3D imaging. The overall information of the lesions is also potentially valuable for predicting their benignity and malignancy. Therefore, the volume of interest in the lesions will be analyzed in a later study. Third, only a single automatic segmentation method from the relevant literature was used in this study. Subsequent studies will explore different automatic segmentation methods to increase the accuracy of model segmentation and further improve model performance.

## 5 Conclusion

The developed DL model can achieve automatic segmentation and automatic classification of BI-RADS 4 lesions in multiview ABVS images with satisfactory performance. This DL model could reduce the number of unnecessary biopsies of BI-RADS 4 lesions without missing any malignant lesions and simplify the workflow for differential diagnosis, indicating its significant potential for clinical applications.

## Data Availability

The raw data supporting the conclusions of this article will be made available by the authors, without undue reservation.
