# Supplementary material for: Multiview deep learning networks based on automated breast volume scanner images for identifying breast cancer in BI-RADS 4
Source: Front Oncol. 2024 Sep 6;14:1399296. doi: 10.3389/fonc.2024.1399296 (PMC11412795; doi:10.3389/fonc.2024.1399296)

Supplementary File 1. Overview of Deep Learning (DL) Models

The Centre 1 and Centre 2 were divided into the training set and independent test set, respectively. We utilized the five-fold cross validation on the training set to optimize the parameters of the models and guide the choice of hyper parameters. The test set was used to evaluate the final model performance independently. A two-stage DL framework consisting of an automatic segmentation module and an automatic classification module has been developed. First, the preprocessed ABVS images were input into the automatic segmentation module for BI-RADS 4 lesion segmentation. Paths were created as the input to the classifier. The classification model was subsequently constructed by CNNs to automatically extract features of the lesions and output the malignant probability. Finally, we visualized and analysed the prediction results of the DL model.

1 Establishment of the automatic segmentation module

1.1 ABVS Images preprocessing

1.1.1 Offline image preprocessing

Histogram equalization and median filtering were used to remove noise from the ABVS images and enhance the image. In addition to the ultrasound imaging portion, the original ABVS images contained useless noise around it such as extensive black boxes and shadow artifacts. So the Sobel operator was utilized as an edge detection method to crop the black box in the ABVS images[1]. The Sobel operator determined the position of the black frame and cropped the black frame based on the weighted

difference of the grayscale of the neighboring points of the pixel point, which was maximized at the content boundary between the black frame and the ultrasound image.

1.1.2 Online image preprocessing

There were 534 (178×3) ABVS images in the training cohort (one each in the axial, coronal and sagittal planes for each case of BI-RADS 4 lesions). Online data augmentation was performed during the training period. The augmentation methods include random cropping the image into 512*512 blocks (if the original image is smaller than 512*512 pixels, randomly fill in zeros around it to reach 512*512 pixels), random mirroring, axial flipping, random lateral flipping, rotating at different angles, random scaling, random affine elasticity transformation, random noise, and so on. Online amplification allows for amplification while training. Since each augmentation was randomized, the amount of data during training was theoretically infinite. The pre-trained model was used on the ImageNet dataset and lesion segmentation was achieved by transfer learning. Notably, the augmented image pixels need to be normalized by the normalization parameters of the pre-trained model before inputting into the segmentation network.

1.2 Establishment of automatic segmentation module

The image segmentation algorithm (DeepLab V3) was introduced by Google, which was extended from V1 and V2. DeepLab V1 is mainly based on the VGG-16 modification, which removed the last two pooling layers and employed Atrous convolution to expand the feeling field. This did not compress the image pixels and preserved the spatial structure information inside the inputable image, improving the

effectiveness of image segmentation. The structural schematic of DeepLab V1 is shown in **S Fig. 1**.

However, DeepLab V1 is weak in multi-scale segmentation. For this reason, DeepLab V2 adds the atrous spatial pyramid pooling (ASPP), a structure similar to Inception, before the final pixel classification in the V1 model. ASPP allows different receptive fields to be obtained by convolving with different expansion rates. The convolution with a low expansion rate has a small receptive field, which can focus on the detailed edge information of the image, and has a better effect on the recognition of small-scale objects; the convolution with a large expansion rate has a large receptive field, which can extract the overall features of the image, and has good effect on the recognition of large-scale objects. Then the fully connected conditional random field (CRF) is used to obtain more accurate segmented images. The ASPP structure is shown in **S Fig. 2**.

In this study, the DeepLab V3 algorithm introduced by Google was used to build the automatic segmentation module for images. DeepLab V3 is a further optimization of the DeepLap V2 model. The DeepLab V3, with the new block5-block7 elongated network structure, adopts an improved atrous spatial pyramid pooling consisting of a 1×1 convolution and three 3×3 convolutions with expansions of (6, 12,18), and finally integrates the feature maps of these four parts with global average pooling with removing fully connected conditional random field. The DeepLab V3 structure is shown in **S Fig. 3**.

2 Establishment of the automatic classification module

The segmented images of the lesion and its surrounding area were as patches to input to the classification module to extract features and automatically output the probability of malignancy. For the reasons that manually labelling masks has a certain degree of subjectivity; the segmentation results of the segmentation model also have certain biases; and the differences between the lesion area and nearby normal tissues may help artificial intelligence classify more accurately. To construct the optimal DL model, we explored the performances of convolutional neural network (CNN) models based on single-view (axial, sagittal, and coronal) and multiview (combined axial, sagittal, and coronal) images, as well as different backbone networks (ResNet50, MobileNet, and Inception-v3) in differentiating benign and malignant BI-RADS 4 lesions. Transfer learning was applied to ensure a strong feature extraction capability. Because of the limited number of samples, pretrained knowledge was effectively applied to a specific task from a mega database such as ImageNet, and the model was then retrained using a small amount of data, which could achieve satisfactory results. Each model was fine-tuned on the dataset of ABVS images to reduce overfitting. The convolutional structure was used as the backbone network, consisting of multiple convolutional layers, average pooling layers, and convolutional modules in series for feature extraction. In the multiview models, each view of the input images corresponds to a backbone network branch, and three branches are concatenated to form the total feature vector.

The third generation GoogLeNet improved Inception-v3 CNN was used to extract the feature of BI-RADS 4 lesions and automatically classify them. Inception-v3 innovatively uses the Inception module instead of the traditional convolutional layer.

The Inception module is divided into three types: Inception A, Inception B and Inception C. Their structures are shown in **S Fig. 4a, 4b** and **4c**, respectively. The exact process of establishing the automatic classification module was described in the main text.

2.1 Parameter and software settings

The construction of the DL model was performed under the open framework Pytorch (https://pytorch.org/) on an A100-SXM4-40GB GPU. In automatic segmentation of lesions, the Adam optimizer was used for model training with an initialized learning rate of 1e-2 batch size set to 8. The maximum training cycle was 200, and at the end of each training cycle, the loss of the latest model on the test set was detected. If the test loss did not decrease in the last 10 cycles (epochs), the learning rate would be divided by 10; if it did not decrease in 40 consecutive cycles, model training would be aborted and the best-performing model on the test set would be saved. Each cycle took about 20 seconds. In automatic lesion classification, Inception-v3 was trained for a maximum of no more than 1000 cycles using the SGD optimizer with an initial base learning rate of 1e-4 and a batch size of 16. If the loss on the test set did not decrease for 30 consecutive cycles, the base learning rate would be divided by 10; if it did not decrease for 90 consecutive cycles, the training process would be aborted. The average time taken per iteration number was 74 seconds.

The weights of the fully connected (FC) layer in the multi-view CNN were initialized according to the Xavier uniform initializer. Additionally, the weights of the Inception-v3 backbone were initialized by applying pre-training weights optimized for the

ImageNet database. For transfer learning, we followed the approach of Azizpour et al. [2] that the entire layer was fine-tuned during the training phase. The multi-view CNN was trained with a batch size of 16. Losses were optimized by Adam with an adaptive learning rate. The CNN was implemented using Keras and trained on the Ubuntu 18.04 system using an Nvidia P6000 GPU.

2.2 Test and Visualization of the DL Model

The fine-tuned parameters were used in the segmentation and classification models of the independent test set to evaluate the effectiveness and the final performance of these models. And the results were analyzed and evaluated by the area under the receiver operating characteristic (ROC) curve (AUC). The larger the value of AUC, the better the ability of this model to recognize malignant BI-RADS 4 lesions. The accuracy, sensitivity, specificity, positive predictive value (PPV), and negative predictive value (NPV) were calculated at the critical value where the Youden index was maximum. The performance of the automatic segmentation network was evaluated by calculating the Dice coefficient (DC). For automatic classification, we computed accuracy, recall, precision, and F1 scores.

Furthermore, gradient-weighted class activation mapping (Grad-CAM) was used on the final convolutional layer of the classification model to visualize the extent to which each region on the ABVS image contributed to the DL model for identifying the malignant BI-RADS 4 lesions. The important regions predicted by the model are highlighted.

The respective meanings and formulas of DC, recall, and precision are given below:

① Recall (R) denotes the probability that a positive sample is correctly predicted. For an m×n segmented image, the formula for recall is expressed as:

$$R = \sum_{i=1}^{m}\sum_{j=1}^{n}\frac{TP}{TP + FN} \qquad (3-1)$$

② Precision (P) indicates the proportion of samples predicted to be in the positive category that are indeed positive, and is expressed by the formula:

$$R = \sum_{i=1}^{m}\sum_{j=1}^{n}\frac{TP}{TP + FP} \qquad (3-2)$$

③ DC is a quantitative index for evaluating the segmentation results, and its formula is expressed as:

$$Dice = \sum_{i=1}^{m}\sum_{j=1}^{n}(2TP/(2TP + FP + FN)) \qquad (3-3)$$

TP is true positive, FP is false positive, FN is false negative, m and n represent the size of the image, and i and j represent the position of the image.

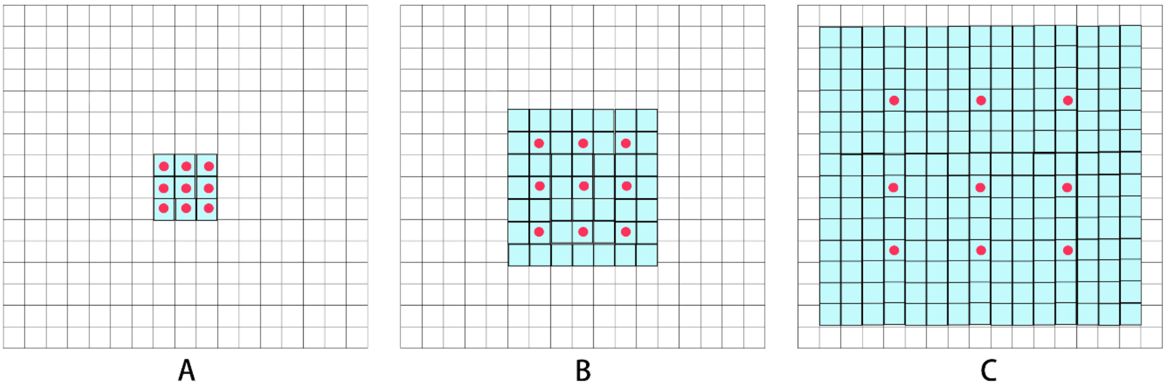

S Fig. 1. The schematic diagram of the atrous convolution

(A) Sensory field with dilation rate of 1; (B) Sensory field with dilation rate of 2; (C) Sensory field with dilation rate of 4.

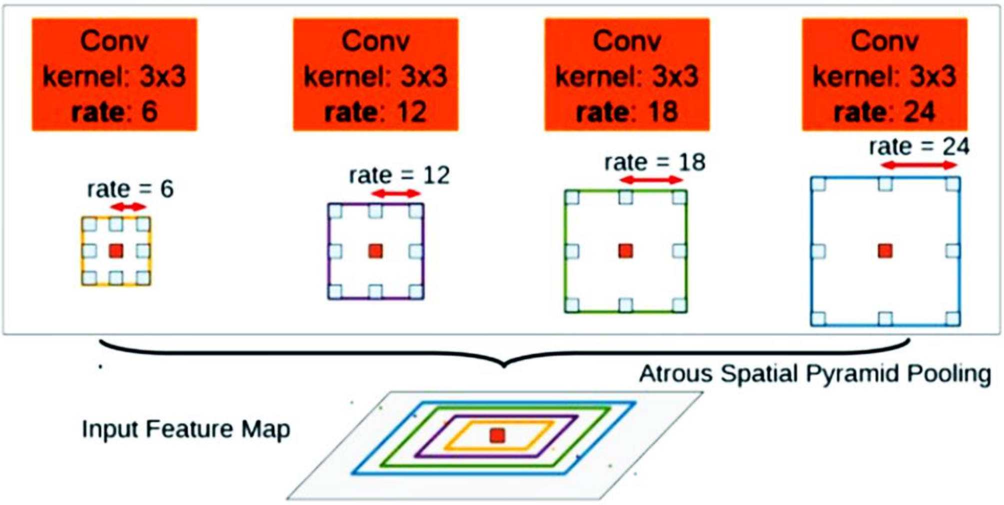

S Fig. 2. The structure schematic diagram of atrous spatial pyramid pooling (ASPP)

To classify the central pixel (the orange color), ASPP extracts multi-scale features by employing multiple parallel filters with different expansion rates, with the effective field-of-view displayed in different colors. (Cited from DeepLabv3: Rethinking Atrous Convolution for Semantic Image Segmentation)

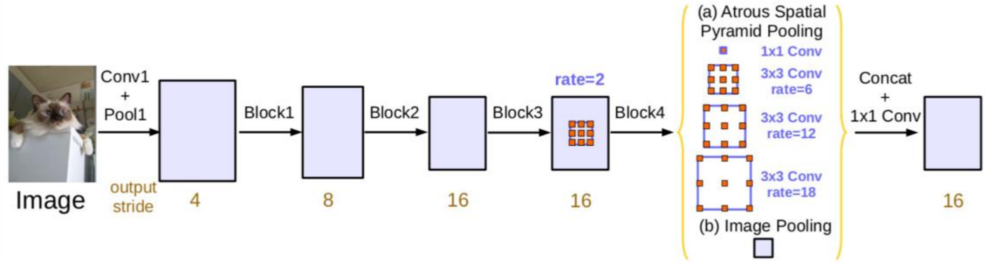

S Fig. 3. The structure schematic diagram of DeepLab V3 (Cited from DeepLabv3: Rethinking Atrous Convolution for Semantic Image Segmentation)

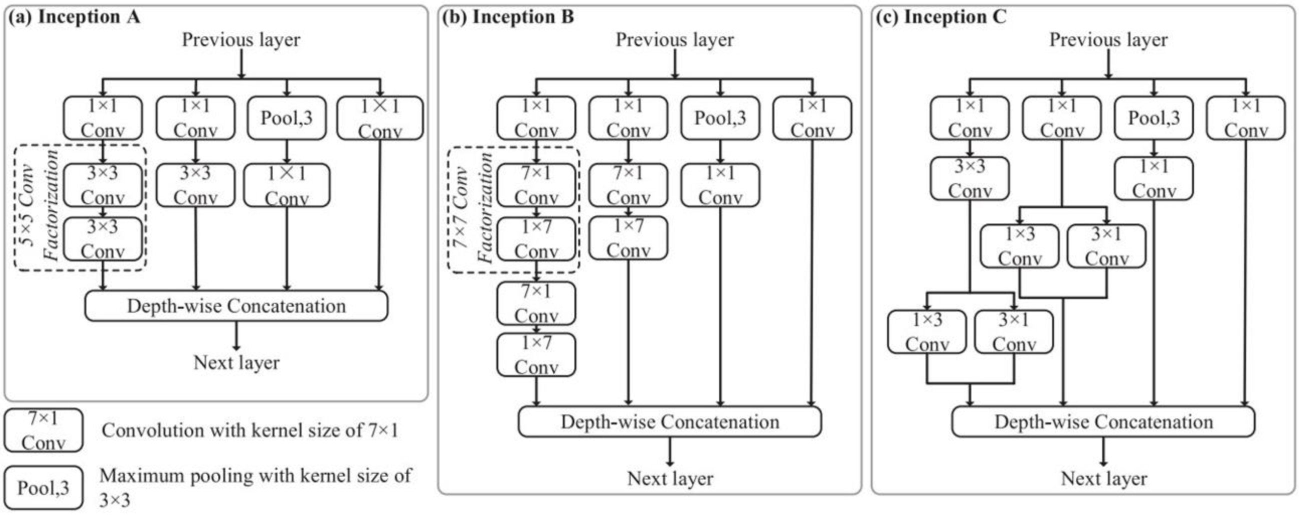

S Fig. 4. Schematic diagram of the structure of Inception A, Inception B and Inception C

Reference:

1. Tang JL, Wang Y, Huang CR, et al (2020) Image edge detection based on singular value feature vector and gradient operator. Math Biosci Eng 17:3721–3735. https://doi.org/10.3934/mbe.2020209

2. Azizpour H, Razavian AS, Sullivan J, et al (2015) From generic to specific deep representations for visual recognition. IEEE Computer Society, pp 36–45

Supplementary File 2. The Dice coefficient curves for automatic segmentation.

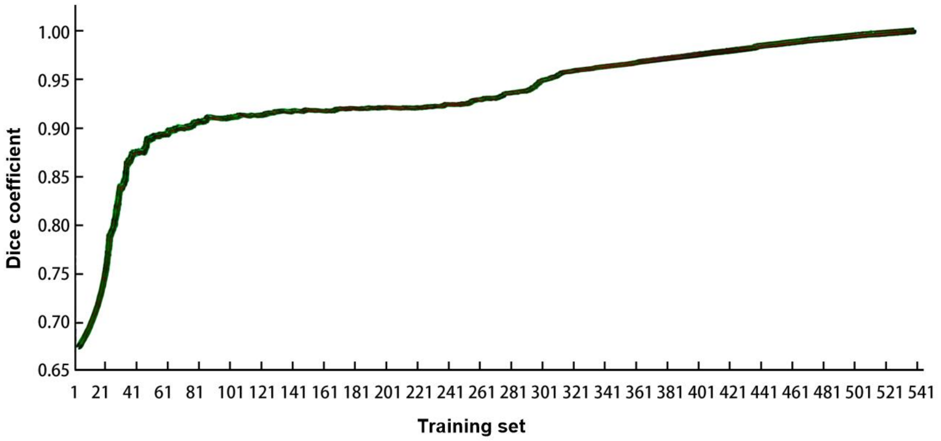

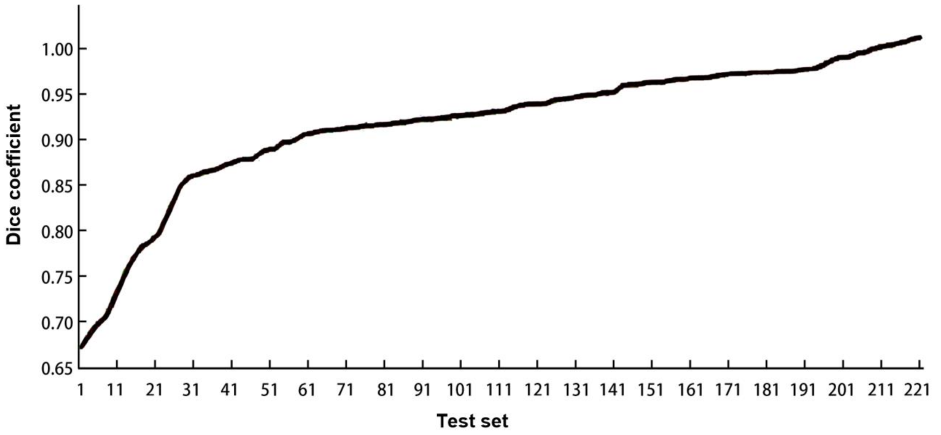

Supplementary File 3. Examples with higher Dice coefficients and lower Dice coefficients.

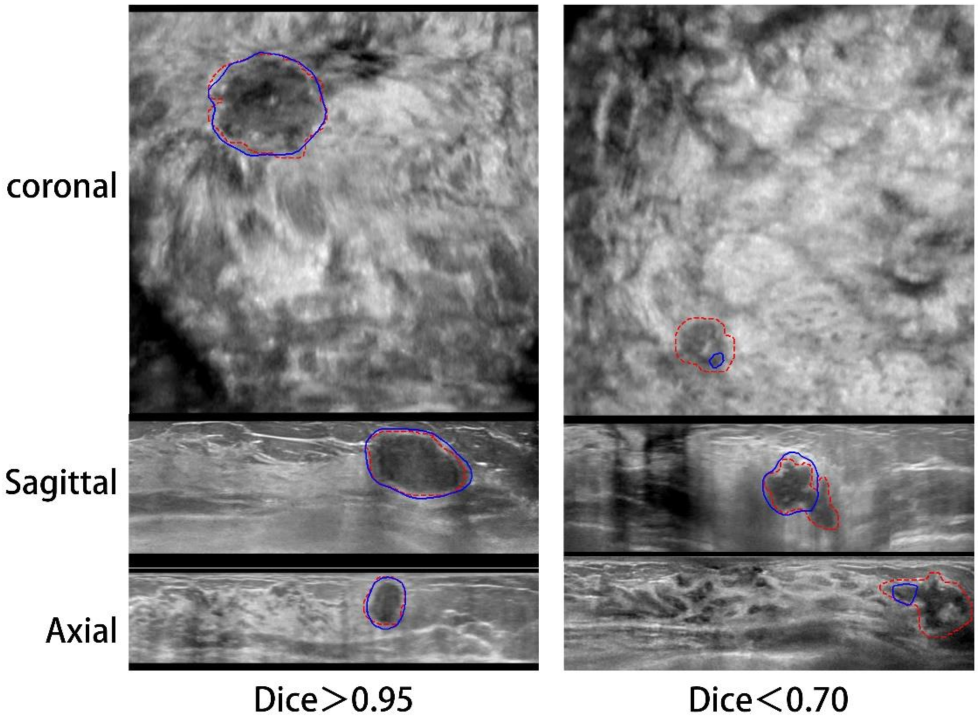

Notes: The ABVS images in coronal, sagittal, and axial views are shown from top to bottom, respectively. The Dice coefficients for the left images are more than 0.95, and the right is less than 0.70. The red dashed line in the images is manually segmented by the doctors, and the blue solid line is automatically segmented by the model.

Supplementary File 4. The diagnostic performance for the training set

(A). The diagnostic performance of multiview models based on different backbone networks for the training set

Backbones	AUC (95%CI)	Sensitivity (%)	Specificity (%)	PPV (%)	NPV (%)
ResNet50	0.936(0.932-0.940)	86.2	81.7	80.8	87.7
**Inception-v3**	**0.957 (0.945-0.969)**	**81.6**	**95.4**	**91.8**	**89.3**
MobileNet	0.901(0.891-0.911)	75.4	87.5	83.8	81.4

(B). The diagnostic performance of single-view and multiview models based on Inception-v3 for the training set

View	AUC (95%CI)	Sensitivity (%)	Specificity (%)	PPV (%)	NPV (%)
Axial view	0.920 (0.913-0.927)	88.2	76.7	85.7	87.5
Sagittal view	0.885 (0.876-0.893)	78.4	82.5	81.6	84.3
Coronal view	0.875 (0.868-0.882)	77.4	80.9	82.8	85.1
**Multiview** *	**0.957 (0.945-0.969)**	**81.6**	**95.4**	**91.8**	**89.3**

Notes: *: Multiview is the combination of axial, sagittal, and coronal planes. The bolded portion is the group with the best indicator.

Abbreviations: AUC: area under the curve; CI: confidence interval; PPV: positive predictive value; NPV: negative predictive value.

Supplementary File 5. The confusion matrix of the Inception-v3-based multiview model with a sensitivity of 100% in the test set.

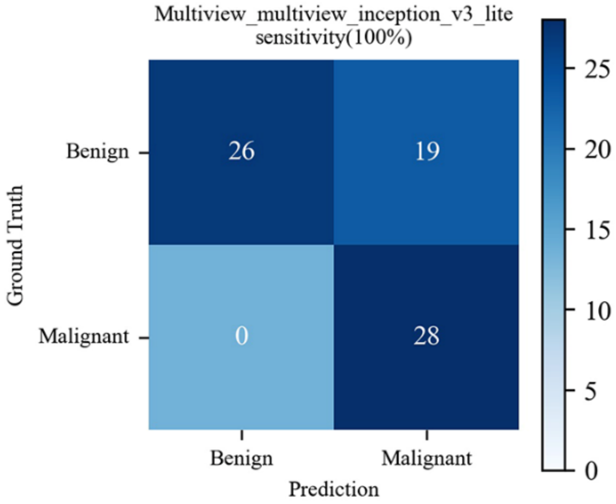

Supplement: Supplementary file 1 [file DataSheet1.pdf]
